# Delayed Enterocutaneous Fistula Following Incisional Hernia Repair Using the Plug-and-Patch Technique: A Report of a Surgically Managed Case

**DOI:** 10.70352/scrj.cr.25-0320

**Published:** 2025-08-19

**Authors:** Kentaro Goto, Masato Narita, Ryoya Yamaoka, Koki Moriyoshi, Hiroaki Hata

**Affiliations:** 1 Department of Surgery, National Hospital Organization, Kyoto Medical Center, Kyoto, Kyoto, Japan; 2 Division of Gastrointestinal Surgery, Department of Surgery, Kyoto University, Kyoto, Kyoto, Japan; 3 Department of Surgery, Kobe City Medical Center General Hospital, Kobe, Hyogo, Japan; 4 Department of Gastrointestinal Surgery, Takashima Municipal Hospital, Takashima, Shiga, Japan; 5 Department of Diagnostic Pathology, National Hospital Organization, Kyoto Medical Center, Kyoto, Kyoto, Japan

**Keywords:** ileal cutaneous fistula, mesh erosion, mesh plug repair, Millikan procedure, plug-and-patch, surgical site infection, tension-free repair, ventral hernia

## Abstract

**INTRODUCTION:**

For inguinal hernia repair, the plug-and-patch technique is commonly employed. Although abdominal wall hernias are occasionally treated with plugs, their safety remains uncertain. Herein, we report a surgical case of enterocutaneous fistula occurring 18 years after incisional hernia repair using the plug-and-patch technique.

**CASE PRESENTATION:**

An 89-year-old woman presented with right lower abdominal discomfort and a skin ulcer and was admitted to our hospital. She had undergone an open appendectomy 64 years prior, followed by incisional hernia incarceration (leading to small intestinal resection) and suture hernia repair 20 years prior. The incisional hernia recurred 2 years postoperatively and was repaired using a mesh. Physical examination revealed a skin ulcer with purulent discharge and erythema on the right lower abdomen surrounding the surgical wound. Contrast-enhanced CT revealed an enterocutaneous fistula. Fasting, drainage, and antibiotic therapy were required before surgery. Laparoscopic resection of the intestinal loop involving the enterocutaneous fistula and the entire mesh was performed. The fascia was closed without a mesh. No hernia recurrence was observed after 39 months.

**CONCLUSIONS:**

The use of plugs is simple but might not be suitable for incisional hernia repair.

## Abbreviations

CRP
: C-reactive protein
NPWT
: negative pressure wound therapy
PVDF
: polyvinylidene fluoride
WBC
: white blood cell

## INTRODUCTION

Since the report by Rutkow and Robbins in 1993,^1)^ the plug-and-patch technique has been widely adopted for hernia repair because of its simplicity and ease of learning.^2)^ Although plugs have occasionally been used to treat abdominal wall hernias,^3)^ their safety remains unknown. We herein report a case of delayed enterocutaneous fistula following incisional hernia repair using the plug-and-patch technique.

## CASE PRESENTATION

**Fig. 1:**
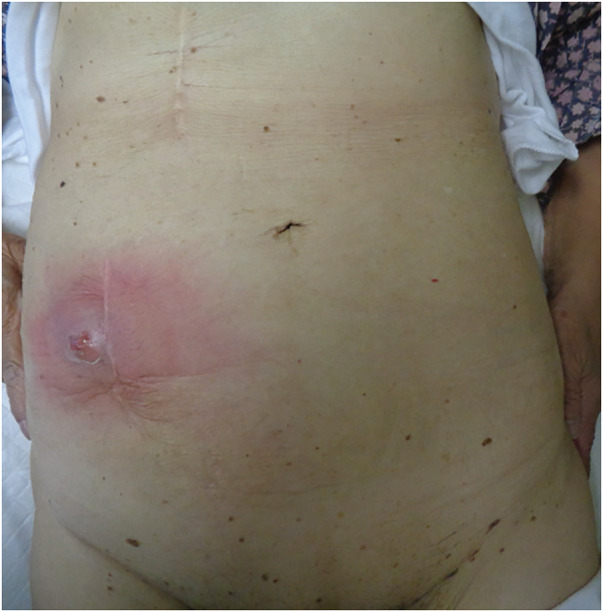
Physical examination at admission. A skin ulcer with erythema was evident on the right lower abdomen outside the surgical wound, with pus discharge from the apex of the ulcer.

**Fig. 2:**
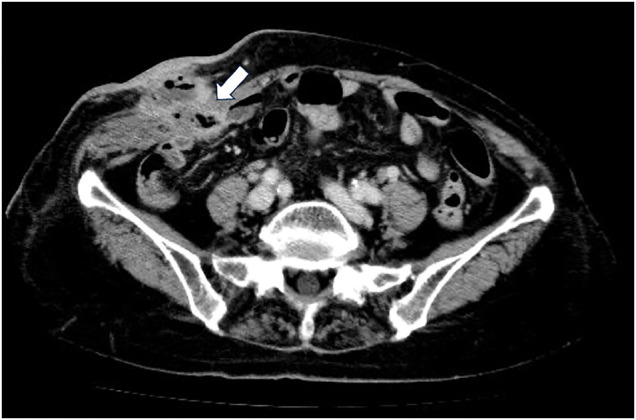
Contrast-enhanced CT findings at the time of the visit. The right lower abdominal wall and skin showed a low-absorption zone with a marginal contrast effect. The ileum extended continuously from this area (white arrow).

An 89-year-old woman presented to our hospital with right lower abdominal discomfort and a skin ulcer. The patient had a history of open appendicectomy 64 years prior, followed by mesh-free incisional hernia repair with partial small intestine resection due to incarceration 20 years prior. An incisional hernia recurred 2 years postoperatively, necessitating mesh repair. The specific details of the surgical procedure and type of mesh used were unclear. On physical examination, the body temperature was 36.7°C, and the patient had hyperkyphosis. There was a skin ulcer on the lateral side of the surgical wound on the right lower abdomen, with pus discharge from the apex of the ulcer and surrounding erythema (**Fig. 1**). Blood tests indicated a mild inflammatory reaction, with a white blood cell (WBC) count of 7800/μL and a C-reactive protein (CRP) level of 2.89 mg/dL. Contrast-enhanced CT revealed a low-absorption area with a marginal contrast effect on the right lower abdominal wall and skin, with continuous extension to the ileum (**Fig. 2**). The fascial defect measured 24 × 17 mm.

The patient was diagnosed with an enterocutaneous fistula caused by mesh migration. Incision and drainage were performed under local anesthesia. We planned to perform surgical intervention after infection management.

**Fig. 3:**
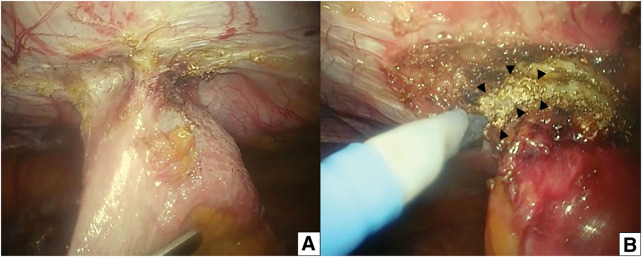
Laparoscopic intraoperative findings. (**A**) Adhesiolysis revealed a dense adhesion of the ileum to the abdominal wall (upper side). (**B**) The ileum and the abdominal wall were sharply dissected, and a plug (black arrowheads) was exposed. The plug appears as frayed fibrous material at the indicated site.

After hospitalization, fasting, enterocutaneous fistula site cleansing, and antibiotic therapy improved the inflammatory reaction of the patient, resulting in a WBC count of 5100 cells/μL, a CRP level of 0.25 mg/dL, and an afebrile state. One week after hospitalization, laparoscopic examination of the umbilical region revealed adhesions to the abdominal wall at the enterocutaneous fistula site. Adhesiolysis revealed a dense adhesion of the ileum to the abdominal wall (**Fig. 3A**). The ileum and the abdominal wall were sharply dissected, and a plug was exposed (**Fig. 3B**). The plug was then divided between the intraperitoneal and abdominal wall sides, and an intestinal resection of approximately 15 cm in length was performed, followed by a functional end-to-end anastomosis. The remaining mesh was completely removed using an open anterior approach. The fascia was closed without additional mesh. Due to inflammation and tissue changes, the anatomical layers were difficult to distinguish. Nevertheless, the closure was performed in a single layer using interrupted monofilament sutures, involving the external oblique aponeurosis and possibly other adjacent layers. Subcutaneous necrotic tissues were removed as much as possible, and negative pressure wound therapy (NPWT) was immediately initiated instead of primary skin closure.

**Fig. 4:**
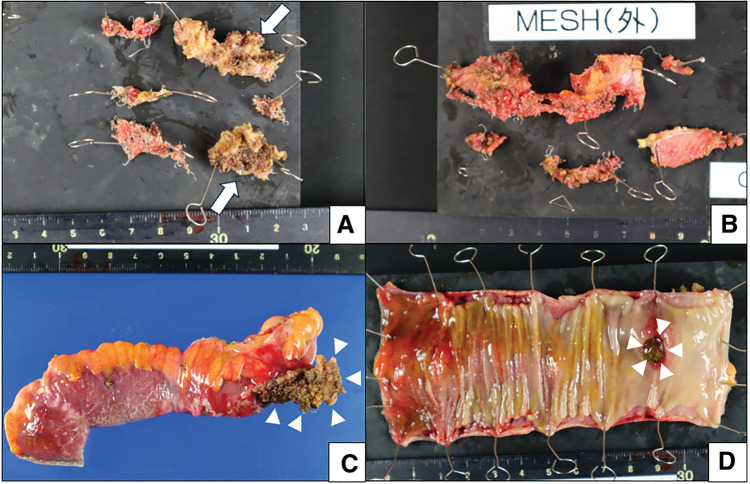
Resected specimens. (**A**) Mesh removed laparoscopically from inside the body cavity and (**B**) mesh removed from the surface of the body wall. The arrows indicate plugs that were removed in a piecemeal fashion. (**C**) Serosal and (**D**) mucosal sides of the resected ileum. The arrowheads indicate the plug penetrating the serosal surface into the luminal side.

**Fig. 5:**
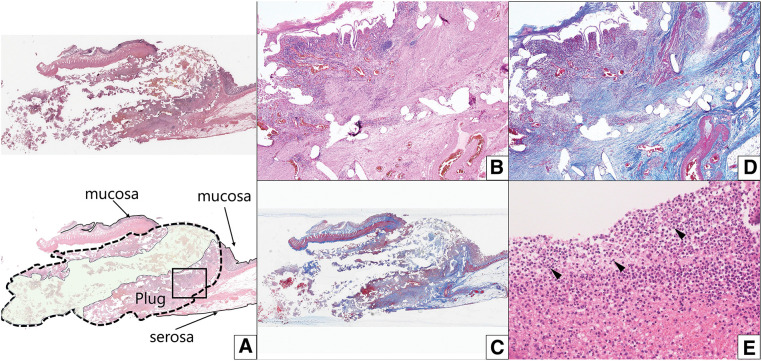
Pathological findings of the resected specimen. (**A**) Hematoxylin–eosin staining and a loupe image of the surgical specimen (×5, upper panel) and its schematic illustration (lower panel), indicating the mucosal and serosal surfaces and the mesh plug (outlined by dashed lines). The upper side of the area surrounding the dashed lines corresponds to the mucosal side, and the lower side corresponds to the serosal side. The plug presents a pointed shape toward the mucosal surface, indicating penetration of the plug from the serosal surface into the mucosal surface. The slit-like area (highlighted in light green) represents exudate and debris accumulated within the cavity of the mesh plug. (**B**) High magnification (×20) of the area outlined by the solid rectangle in (**A**). (**C**) and (**D**) Corresponding Masson’s trichrome staining of (**A**) and (**B**), highlighting fibrosis (blue) inside and outside the plug. (**E**) High magnification (×200) showing exudate with neutrophils (black arrowheads).

In the resected specimen, the plug was exposed in the ileal lumen from the serosa side (**Fig. 4A**–**4D**), indicating that the enterocutaneous fistula was caused by plug migration. The microscopic examination also revealed that the plug had penetrated the ileum and its tip was exposed on the mucosal surface, with some plugs involving the mucosa (**Fig. 5A**). No neoplastic changes were observed. Fibrosis was evident inside and outside the plug, as confirmed by Masson’s trichrome staining (**Fig. 5C** and **5D**), and exudate with neutrophils was present in the slit-like cavity of the plug (**Fig. 5E**).

Repeated wound debridement and lavage procedures were performed. A wound infection at the umbilical site developed on POD 9. The patient was treated with NPWT and promptly improved. The patient was discharged on POD 21. No hernia recurrence or wound infections were observed after 39 months of treatment.

## DISCUSSION

In the present case, the use of a plug for incisional hernia repair resulted in an enterocutaneous fistula. The plug-and-patch technique was introduced as a simple surgical method for inguinal hernia repair^1,2)^ and has been widely adopted, particularly in Japan, as a method with few long-term complications.^4–7)^ Although the plug-and-patch technique was originally indicated only for inguinal hernias, several reports have described its application in incisional or ventral hernia repairs with small orifices.^3,8,9)^ In the present case, the application of the plug-and-patch technique prevented the recurrence of an incisional hernia with a small orifice that had relapsed after primary closure.

Plugs have been used for inguinal hernia repairs; however, they have resulted in a delayed intestinal perforation and enterocutaneous fistula formation.^10–13)^ A recent review determined that migration has been reported in only 26 cases over 29 years among more than 5 million inguinal hernia repairs performed worldwide using the plug-and-patch technique.^14)^ However, this reported frequency may have been underestimated because of a publication bias. Another study showed that mesh migration occurred in 2 (2.6%) of 77 abdominal wall hernias;^15)^ at least in this context, mesh migration would occur following not only inguinal hernia repair but also incisional hernia repair.

We searched the PubMed database using the keywords “plug,” “incisional hernia,” and “migration.” We found only 1 case of delayed intestinal perforation associated with plug use in an incisional hernia.^16)^ This report describes a case of abscess formation involving the small intestine and right hemicolon 12 years after right upper abdominal incisional hernia repair using the plug-and-patch technique. Not surprisingly, no reports have followed up for more than 10 years after incisional hernia repair using the plug-and-patch technique.^3,8,9)^ The late-onset migration can be explained by the adhesion of the intestinal tract to the abdominal wall after surgery. Intermittent or continuous abdominal pressure loading on the intestinal tract would occur if it adheres to the abdominal wall scar. Conceivably, the plug can migrate into the intestinal tract when it is inserted at the point of intestinal tract adhesion. Some reports have suggested that the presence of adhesions from earlier hernia repair might lead to additional adhesions, resulting in migration.^17)^ Additionally, in the present case, the patient had hyperkyphosis, which might have contributed to increased pressure loading on the lower abdomen, potentially leading to plug migration.

In addition to adhesion and pressure loading, the mesh placement method may also cause migration. In this case, plug insertion without considering its configuration might have been a contributing factor. A recent report on chronic postoperative inguinal pain after the plug-and-patch technique revealed that the inside of the plugs was replaced by massive fibrotic tissue, suggesting that a foreign body reaction could render the plugs rigid due to fibrosis.^18)^ Fibrosis potentially transforms the plugs into structures capable of causing intestinal perforation. Furthermore, this study recommended that maintaining the original plug shape is crucial for minimizing fibrosis.^18)^ Presumably, in this case, the large plug would have been packed into the narrow (24 × 17 mm) hernia orifice without considering its original shape, resulting in a decrease in porosity and a change in plug elasticity, which contributed to the progression of fibrosis.^19)^ In inguinal hernias, the Lichtenstein procedure using a plane mesh is more commonly used than the plug-and-patch technique because of its risk of migration.^15)^ Furthermore, international guidelines for inguinal hernia also do not recommend surgery using plugs because of the risk of migration, in addition to the excessive use of foreign materials.^20)^ However, “migrations” may occur not only owing to the inadequate placement of plugs but also because of the inadequate placement of flat mesh. In one case report, a flat mesh used for an abdominal incisional hernia shrank and migrated into adjacent organs.^21)^ This case suggests that even flat meshes can lead to migration if not adequately fixed; therefore, both plug- and flat-type meshes require careful placement to prevent migration.

Another factor was the mesh material used. In this case, a heavy polypropylene plug was applied. Among 77 cases of abdominal wall hernia repair, mesh migration occurred in 2 cases, both associated with the use of a polypropylene mesh.^15)^ A literature review of mesh migration in ventral or incisional hernias revealed that more than half of the cases with migration were attributed to the polypropylene mesh;^22)^ however, some degree of publication bias should be considered, given that the most common material for hernia repair is polypropylene.^23)^ Regarding weight, some reports have demonstrated that using lightweight rather than heavyweight mesh results in less inflammation or oxidative stress.^24)^ This suggests that the weight of the plug and the material could affect migration.

In recent years, alternative materials such as polyvinylidene fluoride (PVDF) have attracted attention owing to their favorable tissue reactions. PVDF meshes reportedly induce less intense foreign body reactions than polypropylene, potentially reducing the risk of fibrosis.^19)^ These materials may offer promising options for improving the safety and durability of hernia repair.

In summary, the use of the plug-and-patch technique for incisional hernia repair may pose a risk of significant complications. Preventing migration may require consideration of the mesh size, weight, and material, as well as the presence of adhesions in the area surrounding the mesh placement site. It is also important to consider the uncertainty of plug fixation and placement and the plug’s shape, which may contribute to erosion into intra-abdominal organs. Greater awareness of the potential dangers of plug-based techniques is essential when considering their use in abdominal wall hernia repair.

## CONCLUSIONS

The use of plugs is simple but might not be suitable for incisional hernia repair.

## ACKNOWLEDGMENTS

We would like to thank Editage (www.editage.com) for the English language editing.

## DECLARATIONS

### Funding

The authors declare that this work did not receive any form of funding.

### Authors’ contributions

KG and MN wrote the manuscript and prepared the figures.

KG and RY performed the surgeries.

KM analyzed pathological data.

All authors reviewed and revised the manuscript, and read and approved the final version.

### Availability of data and materials

All datasets supporting the conclusions of this article are included within the article.

### Ethics approval and consent to participate

Informed consent to participate in this study was obtained from the patient. Patient privacy was considered, and the manuscript includes no identifying information. Our institution does not require ethical approval for case reports.

### Consent for publication

Written informed consent was obtained from the patient for the publication of this case report and any accompanying images.

### Competing interests

The authors declare no competing interests.
